# Supplementary material for: Antenna Protein Clustering In Vitro Unveiled by Fluorescence Correlation Spectroscopy
Source: Int J Mol Sci. 2021 Mar 15;22(6):2969. doi: 10.3390/ijms22062969 (PMC8000295; doi:10.3390/ijms22062969)
Supplement: Supplementary file 1 [file ijms-22-02969-s001.pdf]

Supplementary Materials

Antenna protein clustering *in vitro* unveiled by Fluorescence Correlation Spectroscopy

Aurélie Crepin[1]*, Edel Cunill Semanat[1], Eliška Kuthanová Trsková[1], Erica Belgio[1]*, Radek Kaňa[1,2]

Affiliations
1 Centre Algatech, Institute of Microbiology of the Czech Academy of Sciences, Opatovický mlýn, 379 81 Třeboň, Czech Republic
2 University of South Bohemia in České Budějovice, Faculty of Science, Branišovská 31a, 370 05 České Budějovice, Czech republic

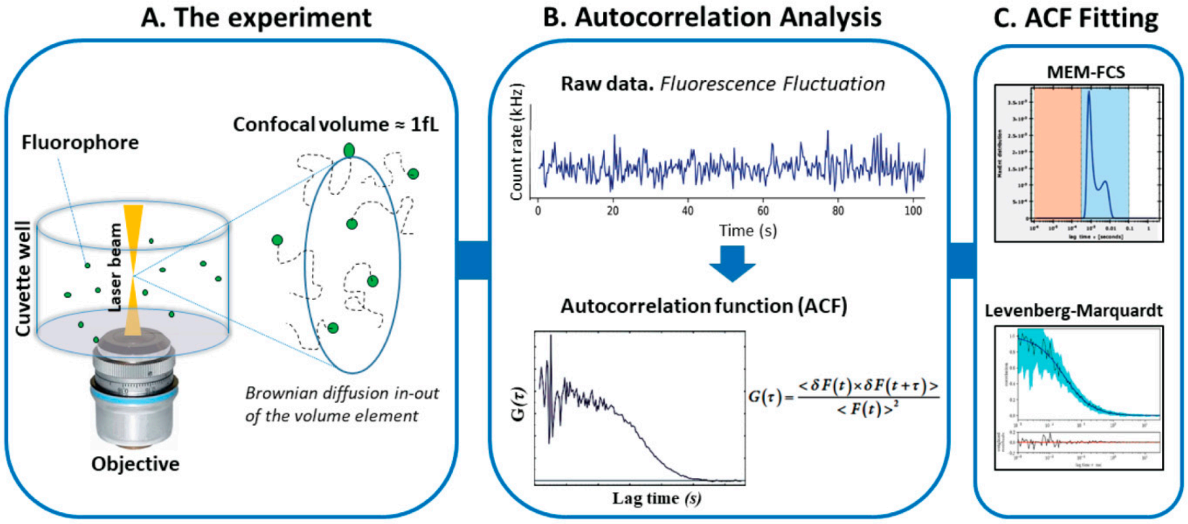

Supplementary Figure 1: Workflow in a typical FCS experiment. (**A**) FCS measurements are usually carried out in very dilute solutions of the analyte of interest, recording fluctuations in the fluorescence signal generated by the entry and exit of molecules in and out the focal volume (~1 fL); (**B**) Typical fluorescence fluctuation intensity traces (top) can be interpreted by means of a fluorescence autocorrelation function (bottom) yielding a fluorescence autocorrelation curve (ACF); (**C**) Fitting ACF with mathematical models allows extracting information on particles molecular properties such as diffusion coefficient, size, oligomeric states, conformational transitions, etc. The models used in the present study are conventional Levenberg-Marquardt and MEMFCS analysis, a model-free fitting method that allows uncovering sample heterogeneity.

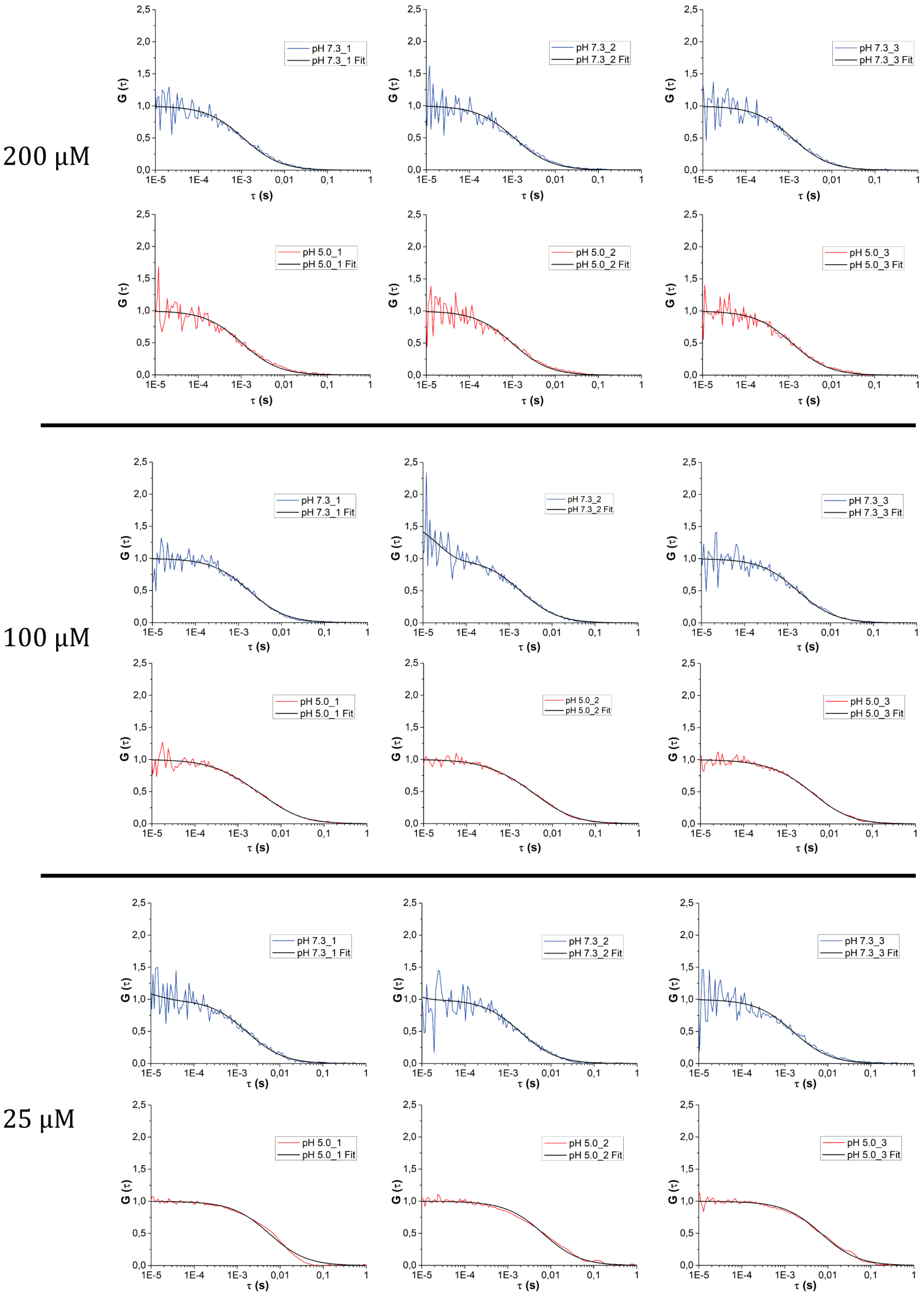

Supplementary Figure 2: Overlap between raw autocorrelation FCS traces (color) and conventional Levenberg-Marquardt fittings (black traces). Designations _1, _2, _3 refer to independent replicates.

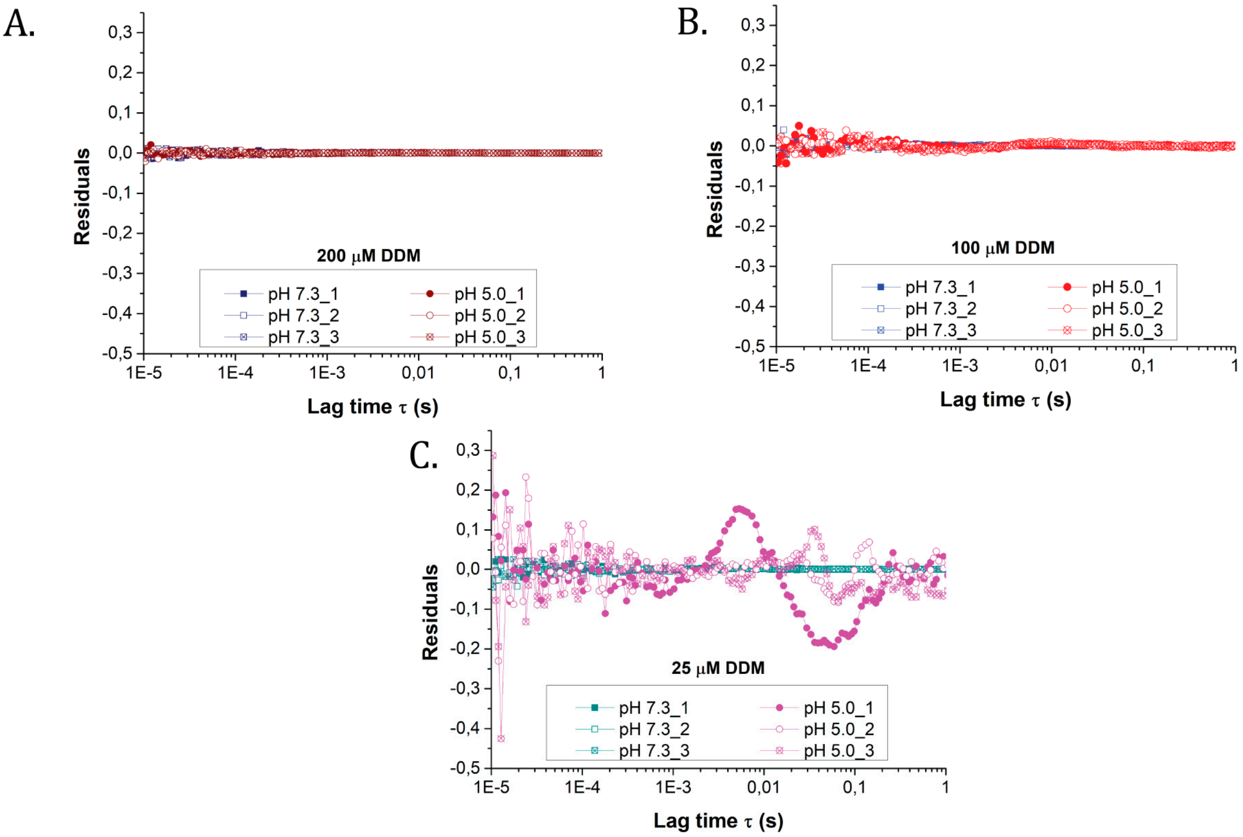

Supplementary Figure 3: Weighted residuals of Levenberg-Marquardt FCS fittings at 200 (**A**) 100 µM (**B**) and 25 µM (**C**). Designations _1, _2, _3 refer to independent replicates.

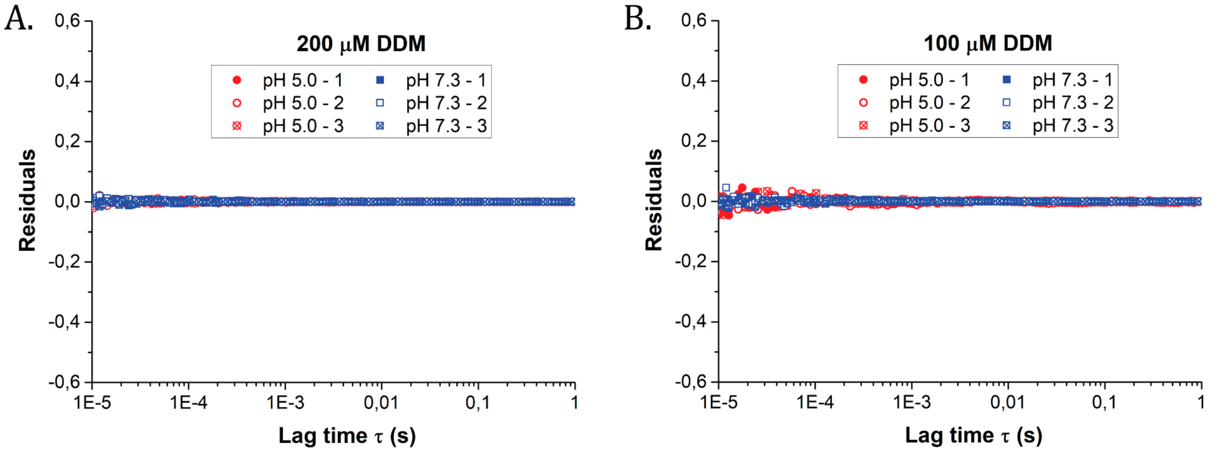

Supplementary Figure 4: Weighted residuals of MEMFCS fits at 200 (**A**) and 100 µM (**B**) using the 3D normal diffusion model, showing the good correlation between the experimental data points and the 3D normal diffusional model. Designations -1, -2, -3 refer to independent replicates.

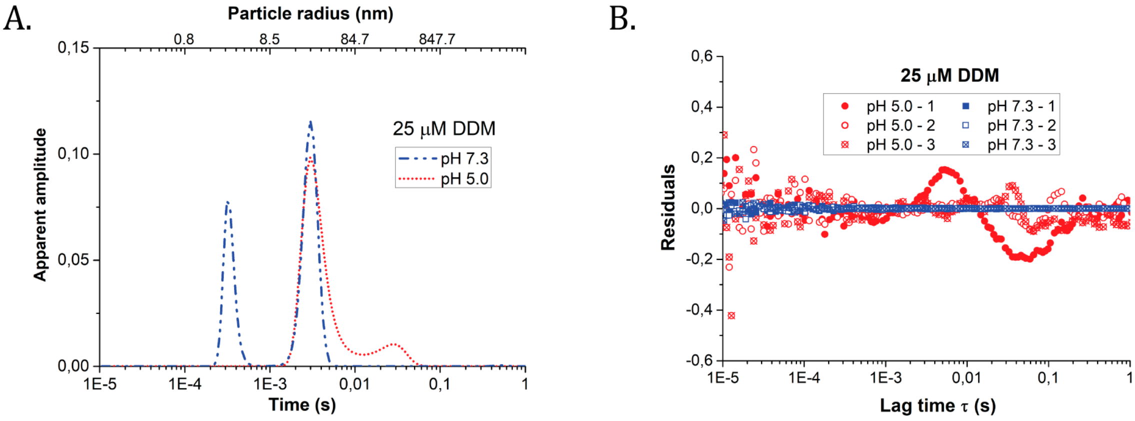

Supplementary Figure 5: FCS results obtained at 25 µM DDM by MEMFCS. (**A**) Typical curves obtained by fitting data at pH 7.3 or pH 5.0. The left-hand peak visible at pH 7.3 displays a lifetime too short to correspond to a diffusion component and more likely represents molecular dynamics; (**B**) Weighted residuals of the MEMFCS fits. The "S-shaped" distribution around the zero value of Residuals, typical of 25 µM pH 5.0 sample, is indicative of low fitting quality. Designations -1, -2, -3 refer to independent replicates.

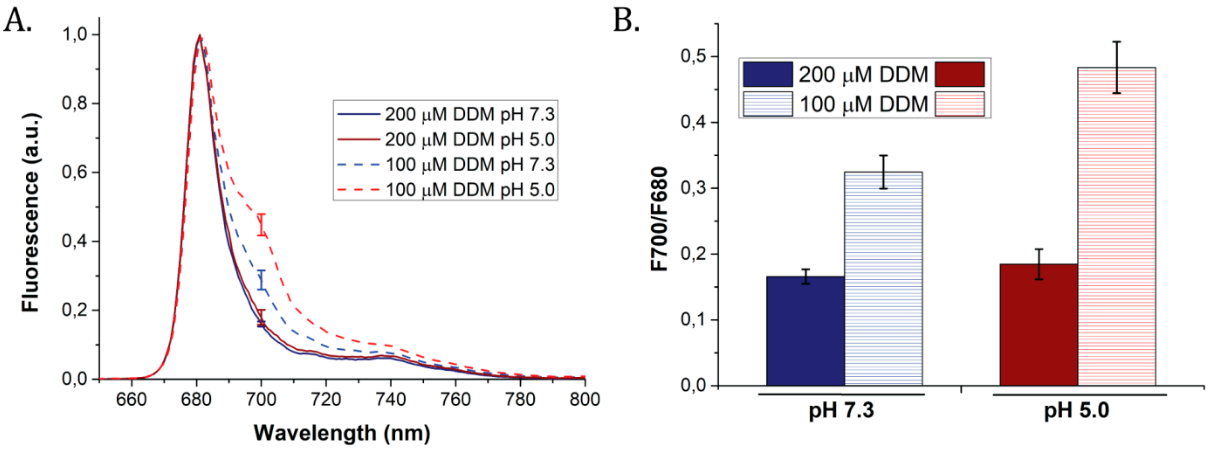

Supplementary Figure 6: 77K fluorescence emission spectra of LHCII clusters formed at higher protein concentration. Purified LHCII were incubated at OD 1.5 at different pH (7.3 or 5.0) and detergent concentration (200, or 100 µM DDM, for protein to detergent ratios of 1:84 and 1:42, respectively). (**A**) 77K fluorescence emission spectra of the clusters obtained. (**B**) Ratio of the fluorescence measured at 700 nm to the fluorescence at 680 nm for each sample. Results are the average of 3 replicates. Error bars: SD.

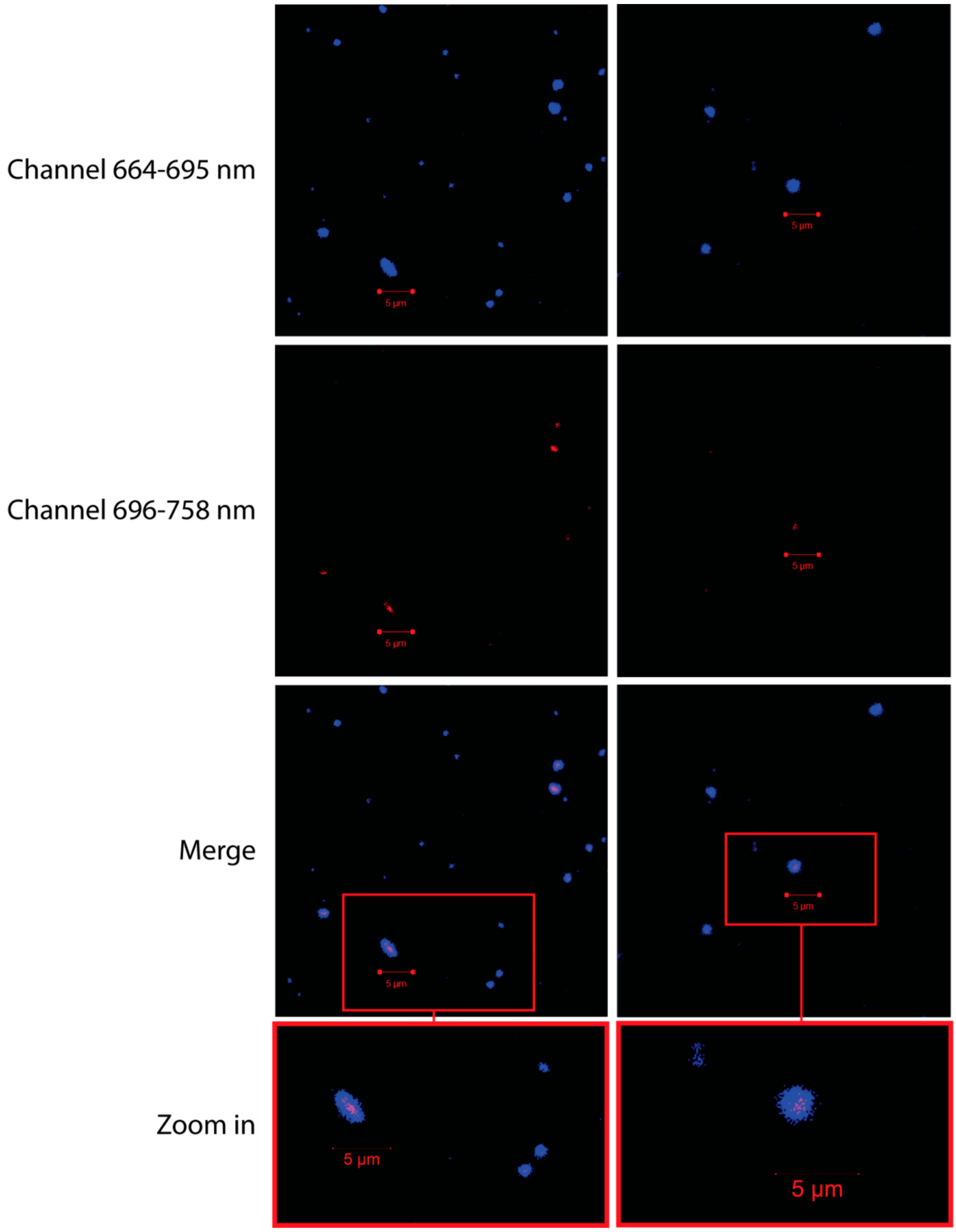

Supplementary Figure 7: Red-shifted fluorescence in cross-sections of large aggregates of LHCII formed at 6 µM DDM and pH 5.0. The emitted fluorescence was measured by confocal microscopy on two channels: 664-695 nm (GaAsP photomultiplier) and 696-724 nm (PMT detector). Pixel dwell time was 1.03 µs. See Methods in the main text for other parameters. Left and right columns represent two examples of cross-sections of aggregates displaying red-shifted fluorescence. Intra-aggregate heterogeneity was observed, with several far-red emitting areas in some large particles.

Supplementary Table 1: Particle size increase, as calculated by conventional FCS (see Figure 2), compared to an increase in the fraction of fluorescence quenched (FQ, calculated from Figure 1 – see Materials and Methods). Percentages are with respect to the 200 µM DDM condition.

	**Size at 200 µM**	**FQ 200 µM**	**Size at 100 µM**	**FQ 100 µM**	**Size at 25 µM**	**FQ 25 µM**
pH 7.3	~10 nm	12 %	~15 nm **(+50%)**	44%	~15 nm **(+50%)**	62%
pH 5.0	~9 nm	20 %	~30 nm **(+400%)***	71%	~65 nm **(+622%)***	76%

* condition displaying sample heterogeneity, yielding imprecise particle size (for more details, see text).
